# The Montreal cognitive assessment (MoCA) 8.1 version, including the memory index score (MoCA-MIS): Italian norms

**DOI:** 10.1007/s10072-025-08066-1

**Published:** 2025-03-17

**Authors:** Caterina Dapor, Maria Devita, Pamela Iannizzi, Elisa Arbia, Angela Bruzzano, Martina Dessì, Domiziana Lupi, Giulia Massa Rolandino, Margherita Rossi, Arianna Saccomano, Elisa Siccardi, Alessia Simonetto, Giulia Vuerich, Sara Zuliani, Konstantinos Priftis

**Affiliations:** 1 Department of General Psychology, University of Padua, Padua, Italy; 2 Geriatric Division, Department of Medicine, University of Padua, Padua, Italy; 3 Hospital Psychology, Veneto Institute of Oncology IOV – IRCCS, Padua, Italy

**Keywords:** Montreal cognitive assessment, MoCA, Memory index score, MIS, Mild cognitive impairment, Dementia, Equivalent scores

## Abstract

**Background:**

We standardized, in Italy, the latest version (i.e., 8.1) of the Montreal Cognitive Assessment (MoCA), including the Memory Index Score (MoCA-MIS), a sensible index of conversion from mild cognitive impairment to dementia.

**Method:**

Six hundred sixty-eight healthy participants took part in the study (age range: 18–99 years, education range: 1–30 years; females: 344). We conducted multiple linear regressions to detect the best predictors (Age, Education, Biological sex, and Cognitive reserve) of participants’ performance.

**Results:**

The results showed that Age, Education, and, occasionally, Biological sex were significant predictors. In contrast, the contribution of Cognitive reserve did not show a systematic pattern. We provided a spreadsheet to precisely transform Raw scores into Adjusted scores for Age, Education, and Biological sex. Finally, Adjusted scores can be classified into Equivalent scores.

**Conclusions:**

We conclude that the present standardization of the MoCA (8.1), including the MoCA-MIS, is a useful contribution for the neuropsychological screening of Italian-speaking persons.

## Introduction

The Montreal Cognitive Assessment (MoCA; [1]) is among the most widely used neuropsychological screening tests for various pathological conditions, such as mild cognitive impairment (MCI), dementia (e.g., Alzheimer’s dementia, Lewy body dementia, fronto-temporal dementia, Parkinson’s disease dementia, Huntington’s disease dementia), amyotrophic lateral sclerosis, multiple sclerosis, epilepsy, and stroke; for a brief review, see [2]. In particular, the MoCA has shown high sensitivity and specificity in identifying MCI [3]. This is likely due to the wide range of cognitive domains that can be investigated, including visuospatial abilities, language, attention, working memory, executive functions, long-term verbal memory, and orientation to space and time.

The instructions for administering and scoring the MoCA (7.1) were originally translated and adapted into Italian by Pirani et al. [4]. Several standardization studies of the MoCA have been conducted in Italy [5–9]. Age, Education, and occasionally Biological sex significantly predicted participants’ performance. Nevertheless, to date, no studies have addressed the standardization of the latest version of the test (i.e., MoCA 8.1; https://mocacognition.com/paper/). Moreover, there are no Italian standardizations, including the MoCA-MIS. We aimed to fill this gap, by providing the standardization of the latest version of the MoCA (i.e., 8.1). For the Italian translation, see [10]; note, however, that this translation was based on that of version 7.1 [4]).

The present study had three main novelty points with respect to the previous Italian standardizations of the MoCA (i.e., 7.1). First, our data were collected from participants from different Italian regions to provide normative data possibly more representative of the whole Italian population (i.e., Veneto, Lombardia, Emilia Romagna, Calabria, Sardegna, etc.). Second, our study incorporated normative data specifically related to the MoCA-MIS, a score derived from the memory subtest of the MoCA. The MoCA-MIS has been found to be particularly sensitive in differentiating amnestic-MCI from normal aging conditions [11] and in identifying people with MCI at high risk of conversion to Alzheimer’s disease [12]. Finally, we included the Cognitive Reserve Questionnaire (CRIq; [13]), among the possible predictors of participants’ performance on the MoCA. The CRIq is a measure of cognitive reserve (CR; [14]). CR has been defined as *“adaptability that helps to explain differential susceptibility of cognitive abilities or day-to-day function to brain aging*, *pathology*, *or insult”* [15]. Individuals with higher CR could exhibit better cognitive performance and/or a slower rate of cognitive decline during the life-span, as compared to those with lower CR [14]. For instance, Montemurro et al. [16] reported that CR outperformed Education as a predictor of participants’ performance on the MoCA.

## Methods

### Participants

We recruited 668 healthy, Italian participants (344 females; age range: 18–99 years, mean age: 56.4 years, *SD* = 21.24 years; education range: 1–30 years; mean education: 13.4 years, *SD* = 4.6 years; Table 1).

**Table 1:**
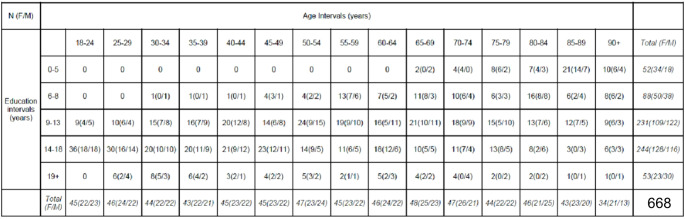
Distribution of the participants as a function of age, education, and biological sex

All participants were recruited, voluntarily, and tested from February to September 2023. Four hundred eighty-eight participants were recruited in northern Italy, 30 participants were recruited in central Italy, and 150 participants were recruited in southern Italy (Table 2).

**Table 2:** Distribution of the participants as a function of the Italian regions of their origin

Northern regions	Participants	Central regions	Participants	Southern regions	Participants
Friuli-Venezia Giulia	123	Lazio	20	Basilicata	3
Lombardia	37	Toscana	7	Calabria	60
Piemonte	38	Umbria	1	Campania	7
Trentino-Alto Adige	2	Abruzzo	1	Puglia	13
Veneto	221	Marche	1	Sardegna	59
Emilia-Romagna	61			Sicilia	8
Liguria	6				
Total	488		30		150

Inclusion criteria consisted of the absence of confirmed diagnosis of neurological and/or psychiatric disorders; no substance abuse; no intake of psychotropic drugs; no other medical conditions possibly affecting cognition (e.g., acquired immunological deficiency syndrome, severe sleep apnea, non-neurological chemotherapy and/or radiotherapy concluded less than one year before recruitment), normal or corrected-to-normal vision and hearing, and an Adjusted score > 26 on the Mini-Mental State Examination [MMSE; 18, 19]. Finally, all participants should have been born in Italy. The study was carried out according to the Declaration of Helsinki and was approved by both the local Ethics Committee and the MoCA Test Inc. (www.mocatest.org). All participants provided their written informed consent to take part in the study.

## Materials

### Anamnestic interview

The anamnestic interview was composed of two sections (Supplemental data 1; https://osf.io/b2eqn/). In the first section, the focus of the examiner was on collecting general information about the participants. For instance, participants were asked to provide their first and last names, date of birth and age, education level, biological sex (male or female), handedness (right-handed, left-handed, or ambidextrous), and professional occupation.

In the second section, detailed information was gathered about the participants’ health status. The goal of the examiner was to determine whether participants were affected by any pathological conditions or if they were taking medications. Initially, the examiner investigated for the presence of visual and/or auditory deficits and whether these were corrected with eye glasses or hearing aids. This ensured that the participant could see and hear well, before being administered the full protocol.

The examiner comprehensively investigated whether participants had any health problems, with particular reference to neurological and psychiatric disorders (e.g., stroke, traumatic brain injury, seizures, depression, anxiety, etc.). The examiner also explored whether participants ever had neurological, psychological, or psychiatric consultations/diagnoses. Participants were also asked if they had ever used or they were currently using drugs and/or abusing alcohol. Finally, participants were asked whether they had trouble sleeping or suffered from insomnia. The main aim of the anamnestic interview was to identify any potential exclusion criteria.

### MoCA

The MoCA (8.1; https://mocacognition.com/paper/) is a paper-and-pencil screening test consisting of a series of subtests administered in the following order: Trail Making Test (TMT), cube copy, clock drawing, denomination, immediate verbal memory recall, forward digit span, backward digit span, letter detection by tapping, serial backward subtraction, two sentences repetition, verbal abstraction, delayed verbal memory recall, and spatial-temporal orientation. Administration lasts for about 10 min and the total score ranges from 0 to 30 points.

We derived the total score (MoCA-TOT) and the scores of the following subdomains, grouped as proposed by Aiello et al. (2022) [5]: Visuo-spatial (MoCA-V: cube copy, clock drawing); Language (MoCA-L: denomination, two-sentence repetition); Attention (MoCA-A: digit span, letter detection by tapping, and serial backward subtraction); Executive functions (MoCA-E: TMT, phonemic fluency, and verbal abstraction); Memory (MoCA-DR: delayed recall) and Orientation (MoCA-O: spatial and temporal orientation).

The MoCA-MIS was also included in the analyses. The MOCA-MIS is derived from the memory subtest. On this subtest, participants were presented with a list of five words and were instructed to remember as many words as possible, both immediately (for two consecutive repetitions) and after a delay of five minutes (i.e., delayed recall subtest). In the delayed recall subtest, in case the participants did not freely recall one or more words, the examiner provided categorical cues (i.e., semantic categories related to the words to be remembered) and, eventually, multiple-choice recognition cues (i.e., a list of three words: the presented word and two distractors). The MoCA-MIS was calculated with reference to the delayed recall subtest. More in detail, the MoCA-MIS was determined by the sum of the words remembered in the delayed recall subtest multiplied by three, plus the words remembered with categorical cues multiplied by two, plus the words recognized through multiple-choice cues multiplied by one (range: 0–15).

### CRIq

The CRIq ([13]; https://www.cognitivereserveindex.org/) is a semi-structured interview developed to evaluate the level of CR of a person. Participants were presented with questions to investigate formal education, job activities, and leisure activities, by reporting the frequency and years of practicing each activity. The total score (CRIq-TOT) provided the overall level of CR [14].

### MMSE

The MMSE is a rapid (10 minutes) paper-and-pencil screening test. The total score ranges from 0 to 30 points. It assesses general cognitive functioning by examining spatial-temporal orientation, immediate and delayed verbal memory recall, attention and calculation, language and visuospatial ability. The MMSE was included to rule out the presence of cognitive impairment, according to the administration and scoring guidelines provided by Foderaro et al. [17, 18].

It is important to acknowledge potential interferences that may arise from administering both the MoCA and the MMSE in the same session. Indeed, the overlap in cognitive domains assessed by the MoCA and MMSE might result in redundant or overlapping information, potentially influencing participants’ performance and introducing bias into the evaluation process. To mitigate these concerns, the tests were presented in a fixed order, with the MoCA administered first and the MMSE second, spaced out by the administration of the CRIq.

### Procedure

Data collection was performed by trained examiners. Each examiner underwent the official training provided on the MoCA website (https://mocacognition.com/training-certification/), obtaining the related certificate. The training was then further completed through additional videos, showing the correct procedure of administration of each part of the protocol (i.e., anamnestic interview; MoCA, CRIq, MMSE). Several simulations and training sessions were also run to ensure that each examiner had correctly learned the procedure. During data gathering, systematic controls were scheduled to ensure a continuous and rigorous oversight by expert supervisors, who personally validated each research protocol and the integrity of the entire procedure.

Each participant was evaluated, alone and in a quiet room, in a single session lasting about 40 min. Each session started with participants giving their written informed consent to take part in the study. Then, participants received the anamnestic interview (Supplemental data 1; https://osf.io/b2eqn/) to evaluate whether all inclusion criteria had been correctly met. Subsequently, participants were administered, in the following fixed order, the MoCA (8.1), the CRIq [13], and the MMSE [17, 18]. The examiner read aloud all the instructions to the participants. Participants’ responses were also audio-recorded, for the examiner to control offline the correctness and completeness regarding the coding and scoring of the participants’ answers.

### Plan of the statistical analyses

We first tested for the effect of Biological sex on participants’ performance, through *t*-tests for independent measures. Then, we conducted Pearson’s product-moment correlations to determine significant predictors of the participants’ performance (Age: non-transformed, logarithm with base 10, natural logarithm, natural logarithm [100 - Age]; Education: non-transformed, square root, inverse). Correlations were run separately for the MoCA-TOT and for the scores in each subdomain. Thereafter, the best predictors were simultaneously entered into multiple linear regressions, separately for the MoCA-TOT and for the scores in each subdomain. After correcting with Bonferroni, significant predictors were used to compute equations for transforming Raw scores into Adjusted scores. Finally, Adjusted scores were placed in ascending order to be transformed into Equivalent scores (see Results).

## Results

We conducted all statistical analyses with JASP [19]. We performed separate analyses on the total score (MoCA-TOT), on the MoCA-MIS, and on the other subdomains [5]: Visuospatial (MoCA-V), Attention (MoCA-A), Language (MoCA-L), Executive functions (MoCA-E), Delayed recall (MoCA-DR), and Orientation (MoCA-O).

### Descriptive statistics

The descriptive statistics for the MoCA-TOT and the subdomains are reported in Table 3. All eight scores were normally distributed, with skewness and kurtosis statistics within the acceptable range of -2 and + 2 (except MoCA-A: kurtosis = 4.719). All Raw scores are reported in Supplemental data 2 (https://osf.io/b2eqn/).

**Table 3:** Descriptive statistics of MoCA-TOT and subdomains

	MoCA-V	MoCA-A	MoCA-L	MoCA-E	MoCA- DR	MoCA-O	MoCA-MIS	MoCA-TOT
Valid	668	668	668	668	668	668	668	668
*Mdn*	3.000	6.000	4.000	3.000	3.000	6.000	12.000	24.000
*M*	3.066	5.293	4.353	2.587	2.993	5.475	11.648	23.771
*SEM*	0.038	0.040	0.030	0.046	0.061	0.024	0.120	0.160
95% CI Mean Upper	3.141	5.371	4.412	2.677	3.112	5.521	11.884	24.086
95% CI Mean Lower	2.990	5.216	4.295	2.497	2.873	5.428	11.412	23.456
*SD*	0.994	1.023	0.769	1.187	1.575	0.610	3.106	4.143
Skewness	-0.794	-1.916	-1.255	-0.604	-0.449	-0.835	-1.059	-0.960
*SE* of Skewness	0.095	0.095	0.095	0.095	0.095	0.095	0.095	0.095
Kurtosis	-0.316	4.719	1.812	-0.459	-0.787	0.308	0.706	0.807
*SE* of Kurtosis	0.189	0.189	0.189	0.189	0.189	0.189	0.189	0.189
Minimum	0.000	0.000	1.000	0.000	0.000	3.000	1.000	8.000
Maximum	4.000	6.000	5.000	4.000	5.000	6.000	15.000	30.000

Note. TOT: Total; MIS: Memory Index Score; V: Visuospatial; A: Attention, L: Language, E: Executive functions; DR: Delayed recall; O: Orientation

### Inferential statistics

#### T-tests on Biological sex

For MoCA-TOT and all the subdomains, we conducted independent-samples *t*-tests to investigate for significant differences in participants’ performance, depending on Biological sex (Supplemental data 2; https://osf.io/b2eqn/). All differences were not significant except for the subdomains MoCA-V, MoCA-A, and MoCA-MIS. For the MoCA-V and the MoCA-A, males outperformed females. On the contrary, for the MoCA-MIS, females outperformed males.

### Correlations

For all MoCA scores (i.e., TOT and subdomains), we ran Pearson’s product-moment correlations to find the best predictors of the participants’ performance (*Age*: non-transformed, logarithm with base 10, natural logarithm, natural logarithm [100 - Age]; *Education*: non-transformed, square root, inverse). According to the results of previous studies (e.g [8]), for most correlations, the best predictors (i.e., significant and higher Pearson’s *r*) were both Age (natural logarithm [100 - Age]) and Education (squared root). The only exception was for MoCA-DR, in which the best predictors were non-transformed Age and Education (squared root). All correlations are reported in Supplemental data 2 (https://osf.io/b2eqn/).

### Multiple linear regressions with predictors: Age, Education, and Biological sex

For each MoCA score (i.e., MoCA-TOT and subdomains), we performed multiple linear regressions with the best predictors (Age: non-transformed, natural logarithm [100 - Age]; Education: squared root). Furthermore, we considered Biological sex (females vs. males) as a categorical predictor (Supplemental data 2; https://osf.io/b2eqn/). To control for the family-wise error, all *p*s were corrected with Bonferroni (0.05/3 predictors: alpha = 0.017).

For all regression models, the variance inflation factor values were all lower than 5 and the tolerance limits were all higher than 0.02. Thus, the results of these analyses suggested that there were no substantial concerns with multicollinearity. Finally, our regression models were characterized by the independence of errors (Durbin-Watson range = 1.5/2.5).

The results of multiple linear regressions showed that Age (non-transformed or natural logarithm [100 - Age]) and Education (squared root) were significant predictors of all MoCA scores, except for the MoCA-O subdomain, in which only Age was a significant predictor. Finally, Biological sex was not a significant predictor of performance, except for two subdomains: MoCA-A and MoCA-MIS (Supplemental data 2; https://osf.io/b2eqn/). On the basis of the results of multiple linear regressions, we transformed Raw scores into Adjusted scores through the equations reported in Table 4.

**Table 4:** Regression equations for transforming Raw score into Adjusted scores

MoCA scores (TOT and subdomains)	Regression equations
*MoCA-TOT*	Adjusted score = Raw score − 3.485 X (natural logarithm [100 - Age] − 3.608) − 1.914 x (Education squared root − 3.602)
*MoCA-MIS (females)*	Adjusted score = Raw score − 2.681 x (natural logarithm [100 - Age] − 3.608) − 0.594 x (Education squared root − 3.602) − 0.687
*MoCA-MIS (males)*	Adjusted score = Raw score − 2.681 x (natural logarithm [100 - Age] − 3.608) − 0.594 x (Education squared root − 3.602) + 0.687
*MoCA-V*	Adjusted score = Raw score − 0.576 x (natural logarithm [100 - Age] − 3.608) − 0.215 x (Education squared root − 3.602)
*MoCA-A (females)*	Adjusted score = Raw score − 0.459 x (natural logarithm [100 - Age] − 3.608) − 0.352 x (Education squared root − 3.602) + 0.198
*MoCA-A (males)*	Adjusted score = Raw score − 0.459 x (natural logarithm [100 - Age] − 3.608) − 0.352 x (Education squared root − 3.602) − 0.198
*MoCA-L*	Adjusted score = Raw score − 0.344 x (natural logarithm [100 - Age] − 3.608) − 0.297 x (Education squared root − 3.602)
*MoCA-E*	Adjusted score = Raw score − 0.462 x (natural logarithm [100 - Age] − 3.608) − 0.737 x (Education squared root − 3.602)
*MoCA-DR*	Adjusted score = Raw score + 0.043 x (Age [non transformed] − 56.355) − 0.212 x (Education squared root − 3.602)
*MoCA-O*	Adjusted score = Raw score − 0.298 x (natural logarithm [100 - Age] − 3.608)

From Raw scores, we calculated Adjusted scores, through the spreadsheets available in supplemental data 3 (https://osf.io/b2eqn/). The Adjusted scores were then ordered from the smallest to the highest. Finally, we calculated Equivalent scores, by means of this software: Https://egdp.shinyapps.io/tolLimits/ [20] (Supplemental data 4; https://osf.io/b2eqn/). All Equivalent scores are reported in Table 5.

**Table 5:** Equivalent scores for each MoCA score (i.e., MoCA-TOT and subdomains)

ES	MoCA-TOT	MoCA-MIS	MoCA-V
0	≤ 17.947	≤ 5.382	≤ 1.186
1	17.948–20.640 (ITL = 19.803)	5.383–7.604 (ITL = 6.513)	1.187–1.856 (ITL = 1.591)
2	20.641–22.348	7.605–9.808	1.857-2.600
3	22.349–24.085	9.809–11.551	2.601–3.274
4	≥ 24.086	≥ 11.552	≥ 3.275
**ES**	**MoCA-A**	**MoCA-L**	**MoCA-E**
0	≤ 3.329	≤ 2.966	≤ 0.584
1	3.330–4.183 (ITL = 3.857)	2.967-3.600 (ITL = 3.309)	0.585–1.378 (ITL = 1.026)
2	4.184–4.931	3.601–3.862	1.379–2.114
3	4.932–5.491	3.863–4.566	2.115–2.651
4	≥ 5.492	≥ 4.567	≥ 2.652
**ES**	**MoCA-DR**	**MoCA-O**	
0	≤ 0.662	≤ 4.289	
1	0.663–1.521 (ITL = 1.048)	4.290–4.841 (ITL = 4.785)	
2	1.522–2.231	4.842–5.024	
3	2.232–3.110	5.025–5.781	
4	≥ 3.111	≥ 5.782	

Note. ES: Equivalent score. ITL: Inner Tollerance Limit

The range of the Adjusted scores, within each Equivalent score (i.e., 0–4), is reported in Supplemental data 3 (https://osf.io/b2eqn/). Starting from the Raw scores, clinicians can use this spreadsheet to calculate the Adjusted scores (Supplemental data 3; https://osf.io/b2eqn/). Finally, the Adjusted scores can be classified on the scale of the Equivalent scores (range: 0–4; 0 = abnormal performance; 1 = borderline performance; 2–4 = normal performance; [21]). When the same Adjusted score was observed at the threshold between two Equivalent scores, the last observation within the lower Equivalent score was shifted to the next smaller Adjusted score, for Equivalent score 0, but to the next larger Adjusted score for Equivalent scores 1–4 [21]. A video explaining the use of the spreadsheets to calculate Adjusted and Equivalent scores is available (Supplemental video; https://osf.io/b2eqn/*).*

### Multiple linear regressions with predictors: Age, Education, Biological sex, and the CRIq-TOT score

We conducted multiple linear regressions to test whether the CRIq-TOT score was a significant predictor of performance, beyond Age, Education, and Biological sex. To control for the family-wise error all *p*s were corrected with Bonferroni (0.05/4 predictors: alpha = 0.012). These analyses are reported in Supplemental data 5 (https://osf.io/b2eqn/). The CRIq-TOT score was not a significant predictor of performance for the MoCA-TOT score and all subdomains, except for the MoCA-E and the MoCA-DR. Nonetheless, in both cases, the increase of Adjusted R^2^ was minimal. More in detail, for MoCA-E, Adjusted R^2^ was 0.338 without CRIq-TOT score and 0.355 with CRIq-TOT score. Finally, for the MoCA-DR score, Adjusted R^2^ was 0.388 without CRIq-TOT and 0.394 with CRIq-TOT score. Therefore, we did not correct Raw scores for the CRIq-TOT score.

### Construct validity

To test for construct validity we conducted a Pearson’s product-moment correlation between the total Raw scores on the MoCA and on the MMSE. Participants’ performance on the two tests was positively correlated (Table 6).

**Table 6:** Pearson’s correlation between MoCA and MMSE

			*N*	Pearson’s *r*	*p*	Lower 95% CI	Upper 95% CI	Effect size (Fisher’s z)	SE Effect size
MoCA-TOT	-	MMSE-TOT	668	0.592	< 0.001	0.541	0.640	0.681	0.039

## Discussion

We standardized, in Italy, the latest version of the MoCA (i.e., 8.1), including the MoCA-MIS and the other cognitive subdomains (i.e., MoCA-V, MoCA-A, MoCA-L, MoCA-E, MoCA-DR, and MoCA-O). We found that Age, Education, and, occasionally, Biological sex were significant predictors of participants’ performance. Our findings are in accordance with those of the Italian standardizations of the previous version of the MoCA (i.e., 7.1; [5, 6, 8]; but see [5], for some differences among the previous Italian standardizations). Those standardizations included the MoCA-TOT score [5, 6, 8] and scores on various subdomains of the MoCA (e.g [5, 8]), but not the MoCA-MIS. Note, however, that, in agreement with our findings, Kessels et al. [2]) reported that Age, Education, and Biological sex were significant predictors of the MoCA-MIS. Finally, construct validity was supported by the significant positive correlation between the Raw scores on the MoCA and on the MMSE.

Montemurro et al. [16] reported that CR outperformed Education, as a predictor of their participants’ performance on the MoCA-TOT score. By contrast, we found, in most analyses, that CR was not a significant predictor of our participants’ performance. In the only two analyses (i.e., MoCA-E and MoCA-DR) in which the CRIq-TOT score predicted the participants’ performance, the increase of Adjusted R^2^ was minimal. Therefore, we did not adjust the Raw scores for the CRIq-TOT score. Although CR is an important concept in neuropsychological evaluation [22], more studies are required to better understand whether CR systematically outperforms other predictors of the participants’ performance on neuropsychological tests (e.g., Age, Education, and Biological sex).

### Comparison among Italian standardizations

We compared the Equivalent scores yielded in our normative study with those provided by the normative studies of the MoCA (7.1) [5, 6, 8]. With reference to the MoCA-TOT, our Equivalent scores aligned most closely with those of Aiello et al. [5] and least closely with that of Santangelo et al. ([8]; Table 7). To provide a concrete example, if we consider a hypothetical 63-year-old female with 13 years of education, scoring 24 on the MoCA-TOT, this person would obtain an Equivalent score of 4 according to Santangelo et al. (Adjusted score = 22.91), but an Equivalent score of 3 according to Conti et al. (Adjusted score = 21.57), Aiello et al. (Adjusted score: 22.82), and our standardization (Adjusted score = 23.98).

With reference to the MoCA subdomains (Table 8), a precise comparison of Equivalent scores classifications was possible only with the study by Aiello et al. [5]. This is because we adopted the subdomain grouping proposed by Aiello et al., which differs from that used by Santangelo et al. [8], because the latter included the phonemic fluency test also within the MoCA-L subdomain. Finally, Conti et al. [6] did not provide Equivalent scores for the MoCA subdomains. Overall, our classification of Equivalent scores was very similar to that provided by Aiello et al. [5]. Notably, in addition to including the MoCA-MIS standardization, a unique feature of our study is the provision of Equivalent scores for the MoCA-DR, which were absent in Santangelo et al. [8] and only partially addressed by Aiello et al. [5]

The mild variability in the Equivalent score classification, among the different studies, highlights the fact that different standardizations might reflect some differences in population characteristics. In this respect, our study, being the most recent, might potentially be the most representative of the current population. Notably, the proximity of our results to those of Aiello et al. [5] likely reflects the temporal closeness of the two studies compared to those of Santangelo et al. [8] and Conti et al. [6], which are nearly a decade old. Furthermore, Conti et al. [6] focused only on a normative sample aged 60–80 years, making it less generalizable to a younger population. Finally, a significant strength of our study lies in the recruitment of participants from several Italian regions (see Method section), providing a more geographically diverse and inclusive normative sample.

It is important to note, however, that the comparison among the various studies should be interpreted cautiously. Indeed, in the studies by Santangelo et al. [8], Conti et al. [6], and Aiello et al. [5], MoCA (7.1) was standardized, whereas we standardized MoCA (8.1). There are some differences between the two versions of the MoCA (7.1 vs. 8.1). For instance, in version 8.1, there are revised instructions for the verbal fluency and abstraction tasks. Furthermore, the stimuli on the letter tapping task (i.e., letter A) are partially different. Moreover, some instructions -although highly similar- are not exactly the same, in the two versions. Nevertheless, the differences between versions 7.1 and 8.1 are not substantial, and we believe that the overall alignment of our Equivalent score classifications with those of the MoCA (7.1) meaningfully supports the validity of our results.

**Table 7:** Comparison among the Equivalent scores classification of Adjusted scores provided by the different standardization studies on the MoCA-TOT score

MoCA-Total Score				
Equivalent Score	Santangelo et al. (2015)	Conti et al. (2015)	Aiello et al. (2022)	Present study
0	≤ 15.5	≤ 17.362	≤ 18.58	≤ 17.947
1	15.51–18.28	17.363–19.500	18.59–20.69	17.948–20.640
2	18.29–20.25	19.501–21.562	20.7–22.56	20.641–22.348
3	20.26–22.23	21.563–23.361	22.57–24.52	22.349–24.085
4	≥ 22.24	≥ 23.362	≥ 24.53	≥ 24.086

**Table 8:** Comparison among the Equivalent scores classification of Adjusted scores provided by the different standardization studies on the subdomains of the MoCA

	Equivalent Score	Santangelo et al. (2015)	Aiello et al. (2022)	Present study
MoCA-V	0	≤ 0.76	≤ 1.36	≤ 1.186
	1	0.77–1.35	1.37–2.03	1.187–1.856
	2	1.36–1.96	2.04–2.64	1.857-2.600
	3	1.97–2.52	2.65–3.22	2.601–3.274
	4	≥ 2.53	≥ 3.23	≥ 3.275
MoCA-E	0	≤ 0.44	≤ 1.07	≤ 0.584
	1	0.45–1.10	1.08–1.87	0.585–1.378
	2	1.11–1.92	1.88–2.45	1.379–2.114
	3	1.93–2.53	2.46–3.07	2.115–2.651
	4	≥ 2.54	≥ 3.08	≥ 2.652
MoCA-L*	0	≤ 3.08	≤ 2.98	≤ 2.966
	1	3.09–3.87	2.99–3.71	2.967-3.600
	2	3.88–4.42	3.72–4.15	3.601–3.862
	3	4.43–5.12	4.16–4.71	3.863–4.566
	4	≥ 5.13	≥ 4.72	≥ 4.567
MoCA-A	0	≤ 2.42	≤ 3.44	≤ 3.329
	1	2.43–3.56	3.45–4.5	3.330–4.183
	2	3.57–4.46	4.51–5.09	4.184–4.931
	3	4.47–5.21	5.1–5.66	4.932–5.491
	4	≥ 5.22	≥ 5.67	≥ 5.492
MoCA-DR	0	-	-	≤ 0.662
	1	-	≤ 0.45	0.663–1.521
	2	-	0.46–1.28	1.522–2.231
	3	-	1.29–2.29	2.232–3.110
	4	-	≥ 2.3	≥ 3.111
MoCA-O	0	≤ 5.03	≤ 4.92	≤ 4.289
	1	5.04–5.92	4.93–5.84	4.290–4.841
	2	5.93–5.94	5.85–5.93	4.842–5.024
	3	5.95–5.98	5.94–5.96	5.025–5.781
	4	≥ 5.99	≥ 5.97	≥ 5.782

* In Aiello et al. (2022) and in our study, the MoCA-L subdomain included naming and sentence repetition. In Santangelo et al. (2015), the MoCA-L subdomain also included the phonemic fluency

### Limits of the present study

Although the present study could contribute to a robust and clinically relevant assessment of the cognitive profile in the Italian population, some limitations should also be acknowledged. For example, the use of a self-reported questionnaire [13] might have led to methodological biases that, in turn, might have negatively influenced our findings about CR. Nevertheless, to the best of our knowledge, no other tools or instruments are currently available to measure CR.

## Conclusions

The latest version of the MoCA (i.e., 8.1) represents an updated contribution to the neuropsychological evaluation of the Italian population. In addition, we provided the first Italian standardisation of the MoCA-MIS index. It is now well established that tests that measure encoding and cueing sensitivity can be sensitive markers of hippocampal memory deficits of early Alzheimer’s disease [23]. In developing the MoCA, Julayanont et al. [12] found that the MoCA-MIS could distinguish amnestic MCI (aMCI) from normal cognition, in older adults. Consequently, the MoCA-MIS is a good prognostic index of the conversion from MCI to Alzheimer’s dementia.
