# Correction to: The Montreal cognitive assessment (MoCA) 8.1 version, including the memory index score (MoCA-MIS): Italian norms

**DOI:** 10.1007/s10072-026-09241-8

**Published:** 2026-07-21

**Authors:** Caterina Dapor, Maria Devita, Pamela Iannizzi, Elisa Arbia, Angela Bruzzano, Martina Dessì, Domiziana Lupi, Giulia Massa Rolandino, Margherita Rossi, Arianna Saccomano, Elisa Siccardi, Alessia Simonetto, Giulia Vuerich, Sara Zuliani, Konstantinos Priftis

**Affiliations:** 1 Department of General Psychology, University of Padua, Padua, Italy; 2 Geriatric Division, Department of Medicine, University of Padua, Padua, Italy; 3 Hospital Psychology, Veneto Institute of Oncology IOV-IRCCS, Padua, Italy

**Correction to: Neurological Sciences (2025) 46:2581–2589**

10.1007/s10072-025-08066-1

In the original version of this manuscript, there are minor typographical errors in Table 5 (columns MoCA-L and MoCA-E, 5th row).

The correct table is shown below.

Incorrect Table 5

Table 5 Equivalent scores for each MoCA score (i.e., MoCA-TOT and subdomains)

**Table:** 

ES	MoCA-TOT	MoCA-MIS	MoCA-V
0	≤ 17.947	≤ 5.382	≤ 1.186
1	17.948-20.640 (ITL = 19.803)	5.383-7.604 (ITL = 6.513)	1.187-1.856 (ITL = 1.591)
2	20.641-22.348	7.605-9.808	1.857-2.600
3	22.349-24.085	9.809-11.551	2.601-3.274
4	≥ 24.086	≥ 11.552	≥ 3.275
ES	MoCA-A	MoCA-L	MoCA-E
0	≤ 3.329	≤ 2.966	≤ 0.584
1	3.330-4.183 (ITL = 3.857)	2.967-3.600 (ITL = 3.309)	0.585-1.378 (ITL = 1.026)
2	4.184-4.931	3.601-3.862	1.379-2.114
3	4.932-5.491	3.863-4.566	2.115-2.651
4	≥ 5.492	≥ 5.492	≥ 4.567
ES	MoCA-DR	MoCA-O	
0	≤ 0.662	≤ 4.289	
1	0.663-1.521 (ITL = 1.048)	4.290-4.841 (ITL = 4.785)	
2	1.522-2.231	4.842-5.024	
3	2.232-3.110	5.025-5.781	
4	≥ 3.111	≥ 5.782	

**Note.** ES: Equivalent score. ITL: Inner Tollerance Limit

Correct Table 5

Table 5 Equivalent scores for each MoCA score (i.e., MoCA-TOT and subdomains)

**Table:** 

ES	MoCA-TOT	MoCA-MIS	MoCA-V
0	≤ 17.947	≤ 5.382	≤ 1.186
1	17.948-20.640 (ITL = 19.803)	5.383-7.604 (ITL = 6.513)	1.187-1.856 (ITL = 1.591)
2	20.641-22.348	7.605-9.808	1.857-2.600
3	22.349-24.085	9.809-11.551	2.601-3.274
4	≥ 24.086	≥ 11.552	≥ 3.275
ES	MoCA-A	MoCA-L	MoCA-E
0	≤ 3.329	≤ 2.966	≤ 0.584
1	3.330-4.183 (ITL = 3.857)	2.967-3.600 (ITL = 3.309)	0.585-1.378 (ITL = 1.026)
2	4.184-4.931	3.601-3.862	1.379-2.114
3	4.932-5.491	3.863-4.566	2.115-2.651
4	≥ 5.492	≥ 4.567	≥ 2.652
ES	MoCA-DR	MoCA-O	
0	≤ 0.662	≤ 4.289	
1	0.663-1.521 (ITL = 1.048)	4.290-4.841 (ITL = 4.785)	
2	1.522-2.231	4.842-5.024	
3	2.232-3.110	5.025-5.781	
4	≥ 3.111	≥ 5.782	

**Note.** ES: Equivalent score. ITL: Inner Tollerance Limit

The original article has been corrected.

